# *mCherry*-Labeled *Verticillium dahliae* Could Be Utilized to Investigate Its Pathogenicity Process in *Nicotiana benthamiana*

**DOI:** 10.3390/genes9100508

**Published:** 2018-10-18

**Authors:** Xiaofeng Su, Guoqing Lu, Latifur Rehman, Xiaokang Li, Lu Sun, Huiming Guo, Hongmei Cheng

**Affiliations:** 1 Biotechnology Research Institute, Chinese Academy of Agricultural Sciences, Beijing 100081, China; suxiaofeng@caa.cn (X.S.); luguoqing007@163.com (G.L.); latif_ibge@yahoo.com (L.R.); lixiaokang2016@163.com (X.L.); sunny_sunlu@126.com (L.S.); guohuiming@caas.cn (H.G.); 2 Department of Biotechnology, The University of Swabi, Khyber Pakhtunkhwa 23561, Pakistan; 3 College of Life Engineering, Shenyang Institute of Technology, Fushun 113122, China

**Keywords:** *Verticillium dahliae*, *mCherry*, pathogenic process, *Nicotiana benthamiana*

## Abstract

*Verticillium dahliae* is a soil-borne phytopathogenic fungus that causes a destructive vascular wilt, but details of the molecular mechanism behind its pathogenicity are not very clear. Here, we generated a red fluorescent isolate of *V. dahliae* by protoplast transformation to explore its pathogenicity mechanism, including colonization, invasion, and extension in *Nicotiana benthamiana*, using confocal microscopy. The nucleotide sequences of *mCherry* were optimized for fungal expression and cloned into pCT-HM plasmid, which was inserted into *V. dahliae* protoplasts. The transformant (*Vd-m*) shows strong red fluorescence and its phenotype, growth rate, and pathogenicity did not differ significantly from the wild type *V. dahliae* (*Vd-wt*). Between one and three days post inoculation (dpi), the *Vd-m* successfully colonized and invaded epidermal cells of the roots. From four to six dpi, hyphae grew on root wounds and lateral root primordium and entered xylem vessels. From seven to nine dpi, hyphae extended along the surface of the cell wall and massively grew in the xylem vessel of roots. At ten dpi, the *Vd-m* was found in petioles and veins of leaves. Our results distinctly showed the pathway of *V. dahliae* infection and colonization in *N. benthamiana*, and the optimized expression can be used to deepen our understanding of the molecular mechanism of pathogenicity.

## 1. Introduction

Verticillium wilt, caused by *Verticillium dahliae*, is the most dangerous threat to hundreds of dicotyledonous plant species, including a wide range of vegetables, crops and woody plants [1]. As a soil-borne fungus, *V. dahliae* produces abundant microsclerotia to resist adverse conditions and thus survive for many years in soil [2,3]. It is mainly disseminated via infected plant material, but once established in a field, it is further spread by the movement of soil through cultivation, wind, and water [4,5]. Although enormous energy has been spent investigating its pathogenicity mechanism and developing resistant cultivars, there is no effective means to protect and treat agricultural fields against this fungus [6,7,8]. Because annual economic losses can reach nearly 250–310 million United States (US) dollars [9], it is imperative that we better understand the infection process and different developmental stages of *V. dahliae* in order to develop novel control strategies for this devastating disease.

Previous studies have shown that the infection and colonization processes are extremely complicated in field soil [10,11,12]. The microsclerotium can repeatedly germinate and sporulate in a moist environment up to nine times, with higher sporulation when nutrients are present in the soil [3]. The germ tube grows and attaches to the root surface of both resistant and susceptible cultivars [13,14]. Some hyphae can penetrate the roots of susceptible cultivars [15]; then, they rapidly grow and accumulate in the vascular bundles in roots, stems, and leaves of plants, which can block the transport of water and nutrients and result in the stunting, necrosis, wilt, and finally death of the host [11]. The progress of the pathogen through the hosts (e.g., lettuce, *Arabidopsis thaliana*, sunflower, *Cotinus coggygria*, and cotton) can be observed and tracked clearly using fluorescently labeled *V. dahliae* and laser scanning confocal microscopy (LSCM) [12,16,17,18,19].

Fluorescent proteins have been widely used as visual, nondestructive molecular markers to study gene expression and regulation and interactions between target proteins and monitor the interaction between the host and fungus and the effects of the fungus on plants in real time, especially for soil-borne fungi [20,21,22,23,24]. In general, the green fluorescent protein (GFP) from *Aquoria victoria* and red fluorescent protein (DsRed, RFP) from *Discosoma* sp. have been most used as the reporter proteins [25]. The infection route was followed in *Arabidopsis* and barley using *Fusarium* strain transformed with GFP [26]. The GFP screening method was used to assess the interaction between microorganisms and *Dothistroma septosporum* and select fungistatic isolates [27]. *GFP*-expressing *Fusarium oxysporum* was also used to visualize colonization of cabbage roots to detect any differences between resistant and susceptible cultivars [28]. In our previous study, we used a GFP-tagged *stt3* mutant and wild-type *V. dahliae* to study the infection process in *Nicotiana benthamiana* in vivo [29]. *DsRed* gene was stably inserted into *Herbaspirillum seropedicae* and *F. oxysporum* to analyze the interaction with the host [30,31] and into *F. graminearum* to observe the infection process in wheat [32].

On the basis of fungal codon preference, the *mCherry* gene can be codon-optimized by introducing various mutations to the amino acid sequence, which yields a protein that matures more completely and has a stronger red fluorescence [33]. To clearly follow the pathogenic process of *V. dahliae* in *N. benthamiana*, here we (1) optimized the coding sequence (CDS) of *mCherry* in *V. dahliae* and (2) generated an isolate of *V. dahliae* isolate that highly expressed the *mCherry* gene so that (3) we could follow the invasion, colonization, and germination of *V. dahliae* in *N. benthamiana*, and (4) quantified the fungal biomass in different tissues and infection stages.

## 2. Materials and Methods

### 2.1. Plants and Fungal Strain

Seeds of *N. benthamiana* were surface-sterilized and sown on Murashige and Skoog (MS) agar (PhytoTechnology Laboratories, Lenexa, KS, USA). Seedlings with two leaves were individually transplanted into plastic pots with disinfested soil (1:1 peat compost to vermiculite). Subsequently, the seedlings were incubated in the greenhouse (23 ± 2 °C, 75 ± 5% relative humidity, 16 h light/8 h dark) and inoculated with fungal suspension liquid when they had eight leaves.

Wild-type *V. dahliae* V991 (*Vd-wt*), a highly virulent and defoliating pathotype, was obtained from Guiliang Jian at the Institute of Plant Protection, Chinese Academy of Agricultural Sciences (CAAS).

### 2.2. Codon Optimization of mCherry and Construction of Expression Plasmid

The coding sequence of the *mCherry* gene was optimized based on fungal codon bias and synthesized by Oligobio, Beijing, China [34,35]. The sequence of the *TrpC* promoter (*TrpC* P) and *TrpC* terminator (*TrpC* T) from *Aspergillus nidulans* were amplified by two sets of primer pairs (Ptrpc and Ttrpc; see Table 1 for all primers). The optimized CDS of *mCherry* was amplified by primer pair Pmch. For the development of the *mCherry* expression cassette, three fragments (promoter, CDS of *mCherry*, and terminator) were fused by overlap extension polymerase chain reaction (PCR). Plasmid pCT-hyg harboring a hygromycin (Hyg)-resistance cassette (*hpt* gene) was kindly provided by Xiaofeng Dai at the Institute of Food Science and Technology, CAAS. The fused fragment was introduced into pCT-hyg by HindIII and XbaI restriction enzymes, and named pCT-HM.

### 2.3. Fungal Transformation

Protoplasts were prepared and transformed using a modified protocol [36]. Fungal spores were harvested from potato dextrose agar (PDA) using a 40 μm nylon filter and adjusted to 10^6^/mL in a final concentration. After 20 h of culturing in a liquid complete medium (CM) at 25 °C, the mycelia were collected on a filter to eliminate nongerminated spores. Mycelia were then digested with 20 mg/mL of Driselase solution (Sigma Aldrich, St. Louis, MO, USA) in 0.7 M NaCl at 33 °C. After 3 h, the mycelia were collected as before, and the solution with the protoplasts was centrifuged at 4000 rpm for 10 min. The protoplasts were then resuspended in an STC buffer (20% sucrose, 10 mM Tris-HCl pH 8.0, and 50 mM CaCl_2_), and 12 μg pCT-HM was added to 200 μL of this protoplast solution (10^6^/mL) with 60% PEG4000, followed by incubation at 25 °C for 20 h. Protoplasts were then observed for red fluorescence with a laser scanning confocal microscope (LSCM; excitation 555 nm, emission 630 nm; Zeiss LSM 700, Jena, Germany), and the transformants were then transferred to PDA plates with hygromycin B (50 µg/mL).

### 2.4. Screening of mCherry-Labeled Verticillium dahliae (Vd-m)

After one week at 25 °C on the selective medium, fungal transformants were examined by LSCM. Fungal colonies that expressed red fluorescence were further cultured in liquid CM for genomic DNA extraction. Positive transformants (*Vd-m*) for *hpt* and *mCheery* were confirmed by PCR using two pairs of primers, Det-hpt and Det-mch (Table 1). Wild-type *V. dahliae* (*Vd-wt*) and H_2_O were used as negative controls. For assessing the stability of *mCherry*, single-spore isolates of the *Vd-m* strains were subcultured for more than six generations, with LSCM observations and PCR assays of each generation.

### 2.5. Characterization of Vd-m

Spore suspensions (10 µL, 10^6^/mL) of strain *Vd-wt* and *Vd-m* were placed on respective PDA plates and incubated at 25 °C for two weeks. Colony diameters were measured every two days. Moreover, the spore suspension (10^6^ spores/mL) of strain *Vd-wt* and *Vd-m* was cultured in liquid Czapek–Dox at 200 rpm and 25 °C in shaker for one week. Spores were counted every day.

### 2.6. Plant Inoculation

The roots of seedlings with eight leaves were immersed in a suspension of 10^7^ spores/mL of *Vd-m* (or *Vd-wt* as the control) for two min, then plants were replanted into new pots. From eight to ten days post-inoculation (dpi), the disease index of five inoculated seedlings was recorded, and the phenotype was observed after 12 days, as previously described [9]. Three independent replications were completed.

### 2.7. Microscopic Observation of Pathogenic Process

From one to ten dpi, two inoculated seedlings were removed from the soil, and roots were gently washed in distilled water to remove soil and unattached spores. Roots were fixed into 10% agarose and sectioned with a knife blade. The slices were placed on a slide with one drop of distilled water and covered with coverslip for microscopic examination, as described above. All experiments were done three times.

### 2.8. Quantification of Fungal Biomass

In order to investigate the infection process at different stages, the fungal biomass was quantified in terms of DNA level by means of quantitative real time polymerase chain reaction (qRT-PCR) and a 7500 Fast Real Time PCR System (ABI, Waltham, MA, USA) [29,37]. At six, eight, and ten dpi, five inoculated seedlings were processed as previously described. The roots, stems, and leaves were removed and grinded into powder for genomic DNA extraction using the Plant Genomic DNA Kit (DP320, TIANGEN, Beijing, China). Primer pair qRT-VdITS was used to amplify ITS1 and ITS2 of ribosomal DNA (Z29511). The *N. benthamiana* housekeeping gene (*Nbactin*, JQ256516) was amplified using a primer set of qRT-Nbactin as an endogenous control. The primers are listed in Table 1.

### 2.9. Statistical Analysis

To ensure the authenticity and reliability of the data, the experiments were independently completed in three sets. In addition, the data were analyzed using SPSS Statistics 17.0 software (SPSS, Chicago, IL, USA). Significant differences among the treatment groups were determined with a least significant difference (LSD) procedure (*p* < 0.01).

## 3. Results

### 3.1. Optimization of mCherry Gene and Plasmid Construction

**Figure 1:**
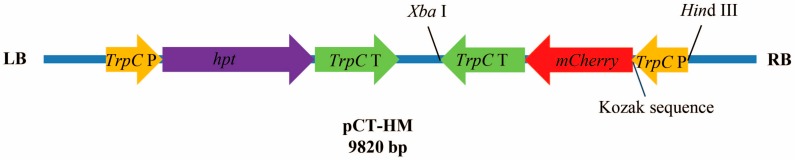
Schematic representation of pCT-HM used for transformation. The *mCherry* and *hpt* genes were under the control of the *TrpC* promoter (*TrpC* P) and terminator (*TrpC* T). P = promoter; T = terminator.

In order to achieve an effective gene expression and strong red fluorescence in *V. dahliae*, we optimized the *mCherry* CDS based on the codon preference of filamentous fungi. The CDS encodes a protein of 236 amino acids (aa), consistent with the original sequence (100% overall identity) (Figure S1). The *mCherry* gene was expressed under the constitutive promoter of *TrpC* from *A. nidulans*, and to stimulate strong expression, the Kozak sequence was also inserted. The *mCherry* expression cassette (1582 bp) was amplified by PCR and introduced into pCT-HM plasmid, which harbors *hpt* as a selection marker (Figure 1).

### 3.2. Confirmation of Vd-m Isolates

**Figure 2:**
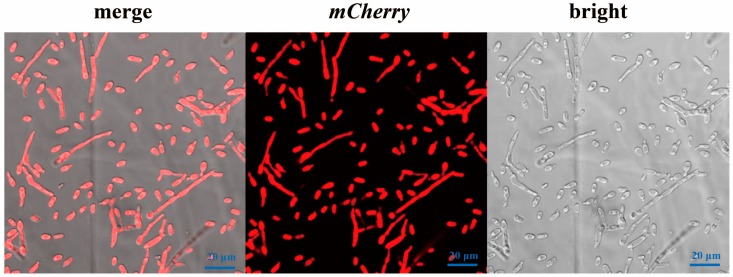
Confocal micrograph of red fluorescence by *mCherry*-labeled *Verticillium dahliae* (*Vd-m*).

After construction of plasmid pCT-HM and protoplast transformation, the *V. dahliae* transformants cultured on PDA with hygromycin were screened using LSCM. The hyphae and spores of five of 46 transformants uniformly displayed strong red fluorescence, and PCR further confirmed the presence of the 549-bp *hpt* fragment and the 427-bp *mCherry* fragment (Figure 2 and Figure S2), indicating a successful integration of exogenous transfer-DNA into the *V. dahliae* genomic DNA. The PCR of wt and H_2_O controls was negative for both fragments. Moreover, stable inheritance of *mCherry* was confirmed by the generation of red fluorescence for six successive rounds of selection. Subsequently, positive transformants with intense red fluorescence were named as *Vd-m* (Figure 2).

### 3.3. Analysis of Biological Characteristics of Vd-m

**Figure 3:**
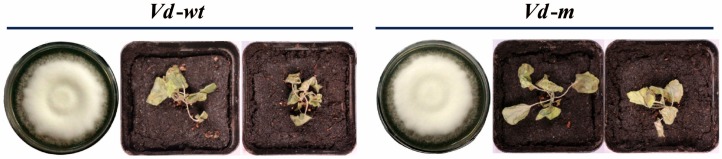
Fungal morphology on potato dextrose agar (PDA) plates and disease symptoms on *N. benthamiana* ten days after inoculation with strain *Vd-m* or *Vd-wt*.

Growth characteristics and disease severity were compared between *Vd-m* and *Vd-wt* (Figure 3 and Figure S3). After two weeks of culture on PDA, the *Vd-m* and *Vd-wt* strains were similar in colony diameter and growth rate (Figure 3 and Figure S3a). When cultured in liquid Czapek–Dox, both strains produced similar numbers of spores on a daily basis (Figure S3b). At ten dpi of *N. benthamiana* seedlings with either *Vd-m* or *Vd-wt* strains, all plants had completely wilted (Figure 3). Moreover, the disease severity at eight toten dpi did not differ obviously (Figure S3c). Overall, our results confirmed that *Vd-m* was similar to *Vd-wt* in its biological characteristics, including pathogenesis, and could be used to study the pathogenic process in *N. benthamiana*.

### 3.4. Attachment and Colonization of Vd-m on Roots

**Figure 4:**
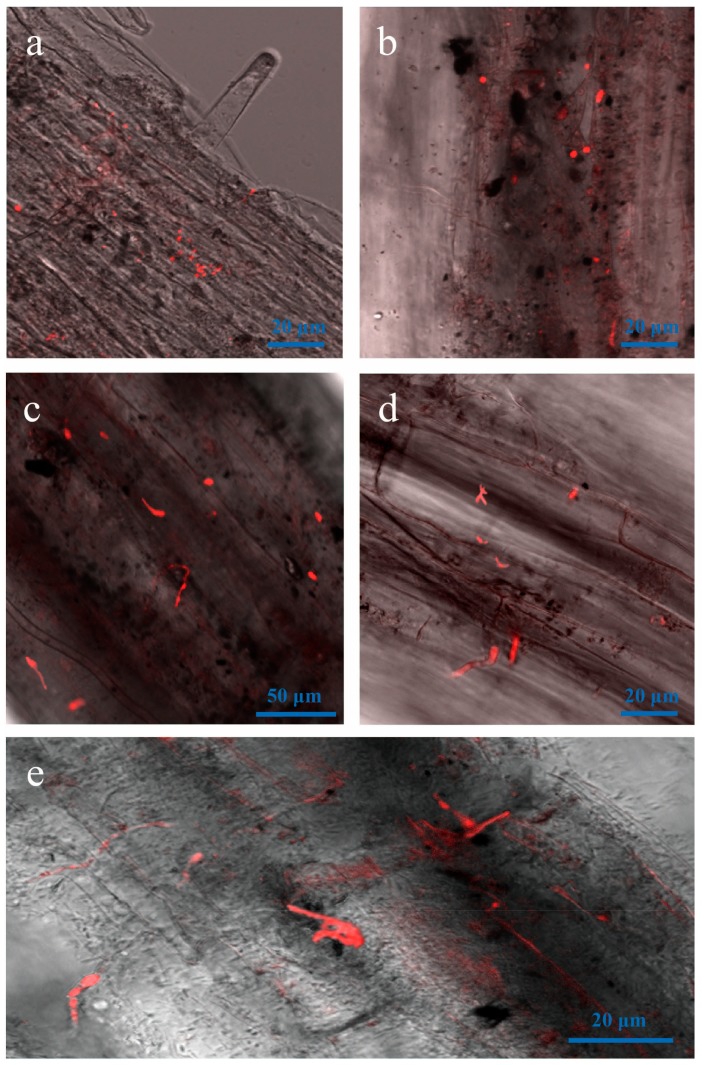
Laser scanning confocal microscope (LSCM) of root surface of *Nicotiana benthamiana* from one to three days after inoculation with the *Vd-m* strain. (**a**,**b**) Spores at one dpi; (**c**,**d**) Germ tubes at two dpi; (**e**) Elongating hyphae at three dpi.

To understand initial infection by *V. dahliae*, we observed roots at one to three days after inoculation with a *Vd-m* strain (Figure 4). At one dpi, spores were present on the root surface at random sites (Figure 4a,b). At two dpi, the spores had begun to germinate, and germ tubes were observed (Figure 4c,d). At three dpi, the germ tubes continued to elongate and had undergone cell division to form hyphae (Figure 4e). By three days after inoculation, *V. dahliae* ended the attachment and colonization process on the root surfaces.

### 3.5. Advanced Penetration Stage of Vd-m in Roots

**Figure 5:**
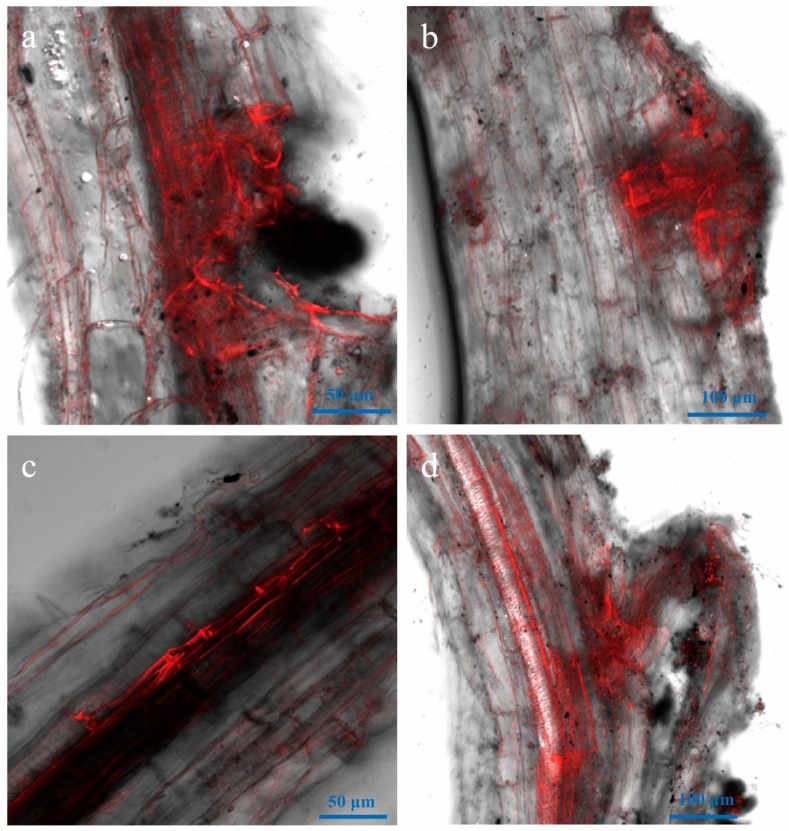
LSCM of hyphal colonization of root vessels from four to six dpi. (**a**,**b**) four dpi; (**c**,**d**) five and six dpi.

By four dpi, hyphal colonization was dense at infection sites, and hyphae had invaded the vascular bundle (Figure 5). Hyphae had also entered the root through wounds and lateral root primordium (Figure 5a,b) and had entered the root xylem vessels and cortical cell junctions (Figure 5c,d). By six dpi, the lower leaves had turned yellow.

### 3.6. Vd-m Extensively Colonized Root Tissues

**Figure 6:**
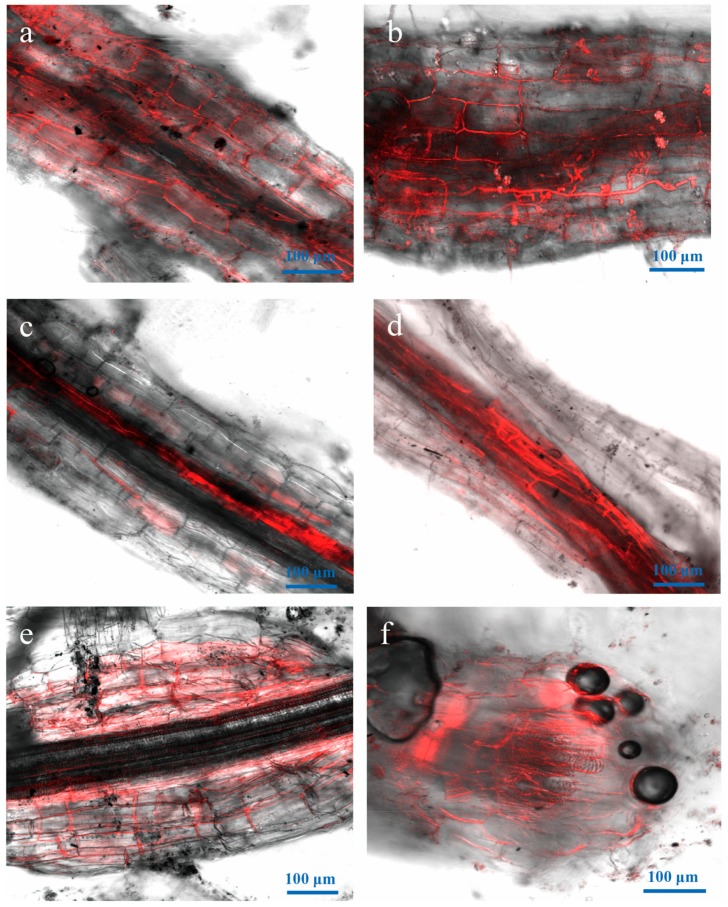
LSCM of colonization inside the root vessels from seven to ten dpi. (**a**,**b**) Extensive hyphal colonization of root tissue and (**c**,**d**) xylem vessels. (**e**,**f**) Cross and longitudinal sections of roots showing extensive hyphal network.

Hyphal colonization of the root gradually increased from seven to ten dpi (Figure 6). Hyphae were growing in the cortex and in the neighboring parenchymal tissues (Figure 6a,b). The hyphae formed a dense network and surrounded the epidermal cell wall. The root xylem vessels were also filled with hyphae (Figure 6c,d). In cross and longitudinal sections, hyphae were clearly observed in the cortex and xylem vessels (Figure 6e,f).

### 3.7. Microscopic Examination of Leaves

**Figure 7:**
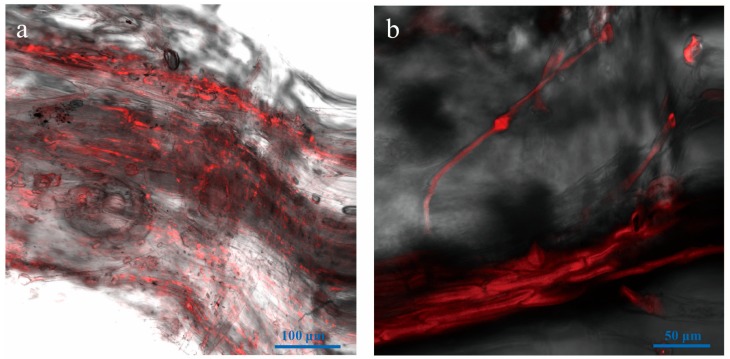
LSCM of hyphal colonization in leaves at ten days after inoculation of roots of *N. benthamiana* with *Vd-m*. (**a**) Leaf petiole and (**b**) veins.

Once *V. dahliae* had colonized the host roots, the hyphae extended through the cortex and xylem vessels of the stem into the leaves. From eight to ten dpi, disease severity and leaf necrosis rapidly increased, and hyphae had spread throughout the plants. By ten dpi, the seedlings had wilted completely. In the leaf petiole, hyphae accumulated extensively (Figure 7a) and grew along the leaf veins, but were not detected in the leaf mesophyll (Figure 7b).

### 3.8. Fungal Biomass at Different Stages

**Figure 8:**
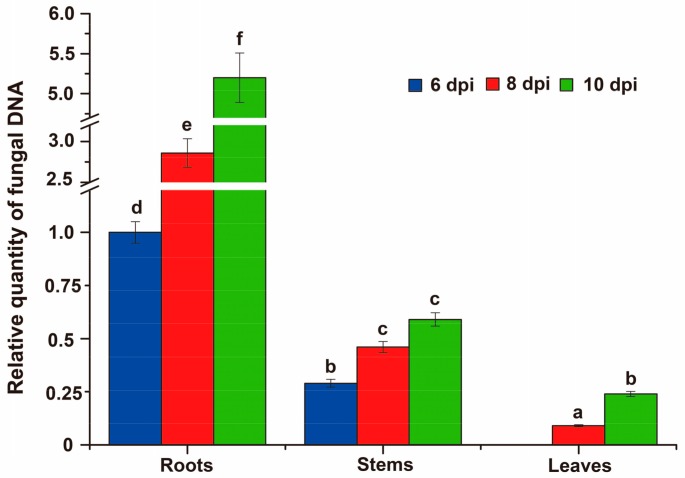
Quantitative real time polymerase chain reaction (qRT-PCR) quantification of fungal DNA extracted from root, stem, or leaves of seedlings of *N. benthamiana* at six, eight, and ten days after inoculation with *Vd-m*. Fungal biomass was determined using ITS1 and ITS2 of *V. dahliae* ribosomal DNA relative to the referenced *Nbactin* gene. Error bars represent standard errors calculated for three independent replicates. Different letters indicate significant differences among the tissues, as determined with a least significant difference (LSD) test (*p* < 0.01).

When we estimated the relative quantity of fungal DNA in roots, stems, and leaves of *N. benthamiana* from six to ten dpi (Figure 8), roots had the most of fungal biomass, followed by stems and leaves. Fungal biomass increased over time in the different tissues, especially in the roots, and reached a maximum at ten dpi. These results were consistent with disease severity and phenotype of the *Vd-m*-inoculated seedlings.

## 4. Discussion

For the expression of green and red fluorescent proteins in fungi to directly observe the pathogenic process [38], a high-efficiency expression system of exogenous genes, including promoter and terminator, requires optimization and selection [39,40]. The CDS of the targeted gene should be optimized according to the codon preference of the species to be transformed [41,42]. An expression cassette, comprising cytidine deaminase, maize-codon optimized Cas9n, and uracil DNA glycosylase, was inserted into *Arabidopsis* to generate gain-of-function mutations [43]. Codon-optimized GFP has been effectively expressed in the cytoplasm and nuclei of *Paramecium caudatum* to visualize protein trafficking and localization [44]. The optimized sequences of genes for reporter molecules GFP, near-infrared fluorescent protein (iRFP), and *mCherry*, were expressed in *Lactoccocus lactis* [45]. In our previous experiment, we constructed RFP-labeled *V. dahliae*, but the red fluorescence was not strong and did not meet experimental requirements because of the lack of codon optimization. Here, thus, we optimized the CDS of *mCherry* and constructed the expression cassette using the *TrpC* promoter and *TrpC* terminator from *A. nidulans*. In addition, we inserted the Kozak sequence, which contributes to the translation of mRNA for the *mCherry* protein.

In a previous study, we developed a protoplast transformation system for *V. dahliae* that is quicker and easier than *Agrobacterium tumefaciens*-mediated transformation [36]. Using the present method for optimization, pCT-HM was introduced into *V. dahliae*. The transformants, primarily screened using LSCM and a selective medium, were confirmed by PCR. After six subcultures of the transformants, we selected one strain for subsequent experiments. Bright red fluorescence was clearly visible from the spores and hyphae. To ensure that the insertion of a foreign gene did not alter the pathogenicity of the *mCherry*-labeled fungus, we compared hyphal growth rate, sporulation abundance, and pathogenicity to the wild type strain to ensure that it did not obviously differ in growth traits, pathogenicity, or virulence. Previous studies have addressed this issue of whether the transformation of a foreign gene has any effect on the pathogenicity of an organism [46,47]. Although virulence was almost the same for eGFP (enhanced Green Fluorescent Protein) transformants and wild-type *Aspergillus carbonarius*, the eGFP transformant produced more ochratoxin A [48]. Similarly, the production of extracellular enzymes and fumonisin B did not differ in the GFP- and DsRed-labeled transformants of *Fusarium verticillioides*, respectively, compared with the wild type [46]. In our study, the insertion of the *mCherry* gene had no significant effect on the virulence of *Vd-m*. Thus, the *Vd-m* strain is suitable for investigating the pathogenicity mechanism in *N. benthamiana*.

As a soil-borne fungus, *V. dahliae* infects the host through the roots [14,49]. However, its infection process in many host plants is still unclear. In a study of infection of cotton, the fungus infected the root tips, and colony density was low on the root surface [50]. Once *V. dahliae* colonized and invaded the root meristematic tissues, the spores began to germinate in the vascular bundles [19]. The hyphae of *V. dahliae* primarily entwine the outside of root hairs tightly by 24 h post inoculation (hpi) and elongate on the root hair surface, extending toward the root surface of *Brassica napus*. No specific infection structures are formed during fungal penetration of the root epidermis [51]. In contrast, by 48 hpi, appressoria had developed on lettuce roots, and the fungus penetrated the adjacent cell of the lettuce root [12]. After spore germination, the apex of some hyphae differentiate into simple penetration structures, hyphopodia, which adhere to the root surface [52]. Penetration pegs are then generated at the bottom of hyphopodia [53]. Hyphae cover the root surface without any apparent preference for a specific part of the root in *Arabidopsis* and cotton [16]. Root hairs, root caps of lateral roots, and root elongation zones of sunflower are covered by *V. dahliae* mycelium between 24 and 96 hpi [17].

In the present study, we used *Vd-m* to monitor the pathogenic process in *N. benthamiana* for the first time. At one dpi, spores on the main and lateral roots were swollen and had begun to germinate on the root surface. As the hyphae elongated, the fungus penetrated the epidermal cells and grew along the longitudinal vessels. Regardless of the part infected and type of growth, hyphae invaded the host quickly through root wounds and lateral root primordium, then intensely aggregated at these sites. After that, the hyphae quickly grew and began to enter into the xylem in roots.

*V. dahliae* hyphae form a network on the epidermal cells and around the cell wall, rather than penetrating the cells [53] as we found here. In addition, hyphae quickly grew in the xylem and filled the vascular tissue in roots, stems, and leaves. In the leaves, hyphae were detected in the petiole and veins, rather than mesophyll tissue. A similar pattern was observed for *Verticillium longisporum*, and fungal DNA and extensive microsclerotia accumulated in the petiole of *Arabidopsis* [54]. In *C. coggygria*, fungal DNA of *V. dahliae* is present in stems by six dpi, in branches by 12 dpi, and leaves by 14 dpi [18]. Compared with stems and leaves, the roots have distinctly more fungal biomass, consistent with the initial colonization of the root and growth into the vascular bundles and subsequent invasion of the stems and leaves [9,16]. In sunflower, *V. dahliae* spreads into the vascular bundles and cortical tissues, leaf veins, and eventually pollens and seeds [17]. Overall, in this systemic disease of the vascular tissue, the spores colonize the root surface and penetrate the epidermal cells. Hyphae predominantly survive in the xylem during the pathogenic process, obtaining nutrients such as sugars, inorganic salts, and amino acids [55].

## 5. Conclusion

In this study, the CDS of *mCherry* was optimized, and the expression cassette was constructed based on *V. dahliae* codon preference. The pCT-HM was introduced into fungal protoplasts to generate *Vd-m* strains that produce a bright red fluorescence. Using LSCM and the *Vd-m* strain, we clearly visualized the infection, colonization, and systemic development of *V. dahliae* in *N. benthamiana*. In future studies, this optimized *mCherry* system could be linked with a targeted gene to facilitate subcellular localization. Moreover, the *Vd-m* strain is exceedingly valuable for further investigating the pathogenic process in detail and fungal interactions with host plants.

## Figures and Tables

**Table 1:** Primers used in this study

Primers	Sequence (5′-3′)
Ptrpc	atc*AAGCTT*TTGAAGGAGCATTTTTGGGCTTGGC
	CTCGCCCTTGGAGACCATGGTGGCATCGATGCTTGGGTAG
Pmch	ATGGTCTCCAAGGGCGAGGAGGACAAC
	CTACTTGTAGAGCTCGTCCATGCCGCC
Ttrpc	CATGGACGAGCTCTACAAGTAGAGTAGATGCCGACCGGGATCC
	aac*TCTAGA*TTATCTTTGCGAACCCAGGGGCTG
qRT-VdITS	CCGCCGGTCCATCAGTCTCTCTGTTTATAC
	CGCCTGCGGGACTCCGATGCGAGCTGTAAC
qRT-Nbactin	GGACCTTTATGGAAACATTGTGCTCAGT
	CCAAGATAGAACCTCCAATCCAGACAC
Det-hpt	GAGGGCGAAGAATCTCGTGCTTTCA
	TGTTATGCGGCCATTGTCCGTCAGG
Det-mch	CTACGTTAAGCACCCCGCCGACATT
	CTGCTCGACAATCGTGTAGTCCTCGT

Underlined sequences were designed based on the *mCherry* coding sequences (CDS) following the overlap extension polymerase chain reaction (PCR). Restriction sites according to the plasmid pCT-hyg are in bold italic letters, and oligonucleotides for enzyme digestion are in lower-case letters.

## Supplementary Materials

The following are available online at http://www.mdpi.com/2073-4425/9/10/508/s1, Figure S1. Alignment and amino acid sequences of *mCherry* sequence. The original and optimized sequences were aligned with the program DNAMAN. Differing nucleotides are represented in red. Figure S2. PCR detection of *hpt* (549 bp) and *mCherry* (427 bp) genes, confirming exogenous tDNA integration into *V. dahliae* genomic DNA. Figure S3. Biological characteristics of *Vd-m* and *Vd-wt*. (a) Colony diameter on PDA plates after three, six, nine, and 12 days. (b) Spore production in shake culture on Czapek–Dox medium after four, five, six and seven days. (c) Disease index of *N. benthamiana* at eight, nine, and 10 dpi.
