# Supplementary figures and images for: mCherry-Labeled Verticillium dahliae Could Be Utilized to Investigate Its Pathogenicity Process in Nicotiana benthamiana
Source: Genes (Basel). 2018 Oct 18;9(10):508. doi: 10.3390/genes9100508 (PMC6210675; doi:10.3390/genes9100508)

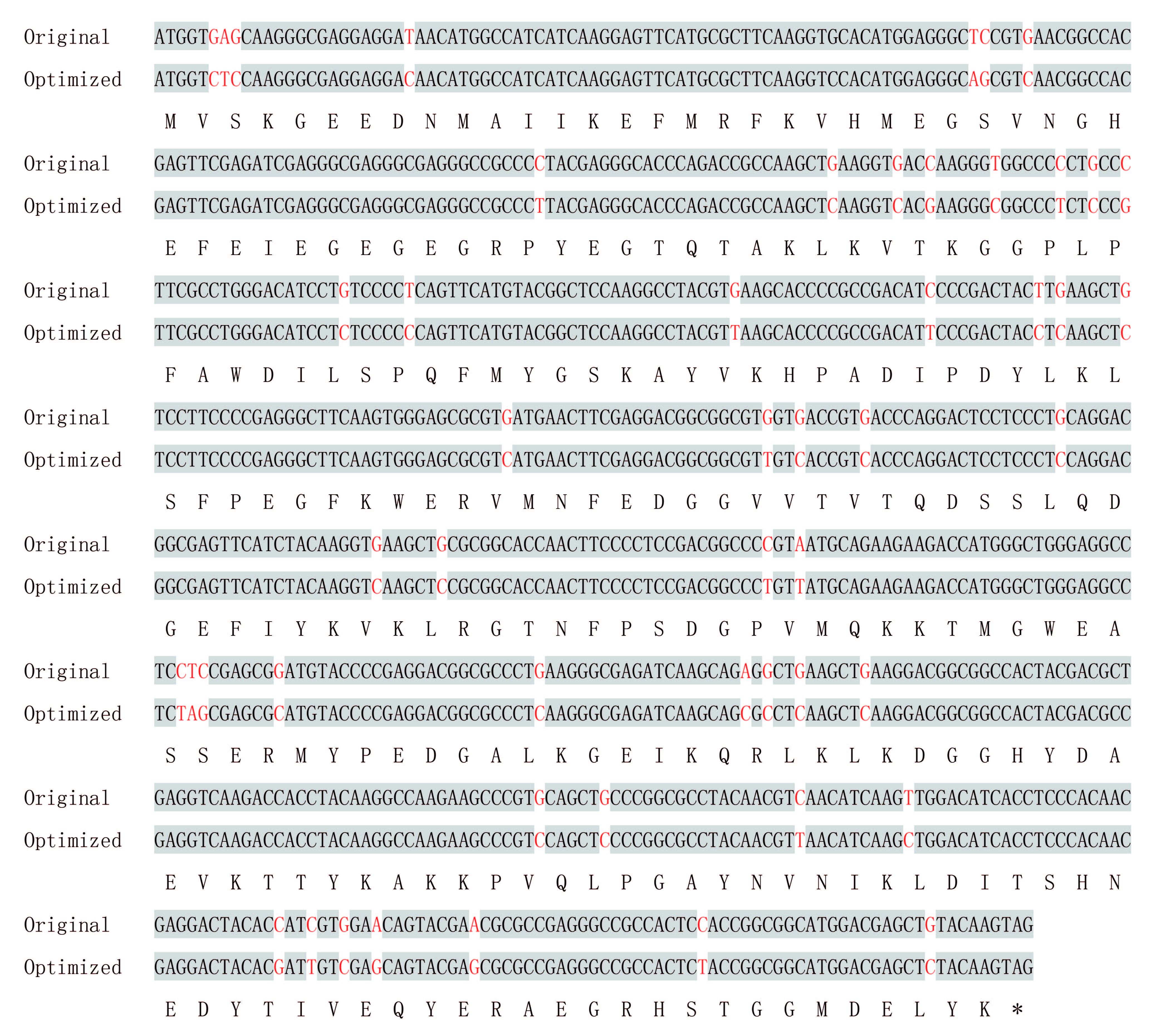

Supplement: Supplementary file 1 [file genes-09-00508-s001.zip › Supplementary File/Fig. S1.jpg]

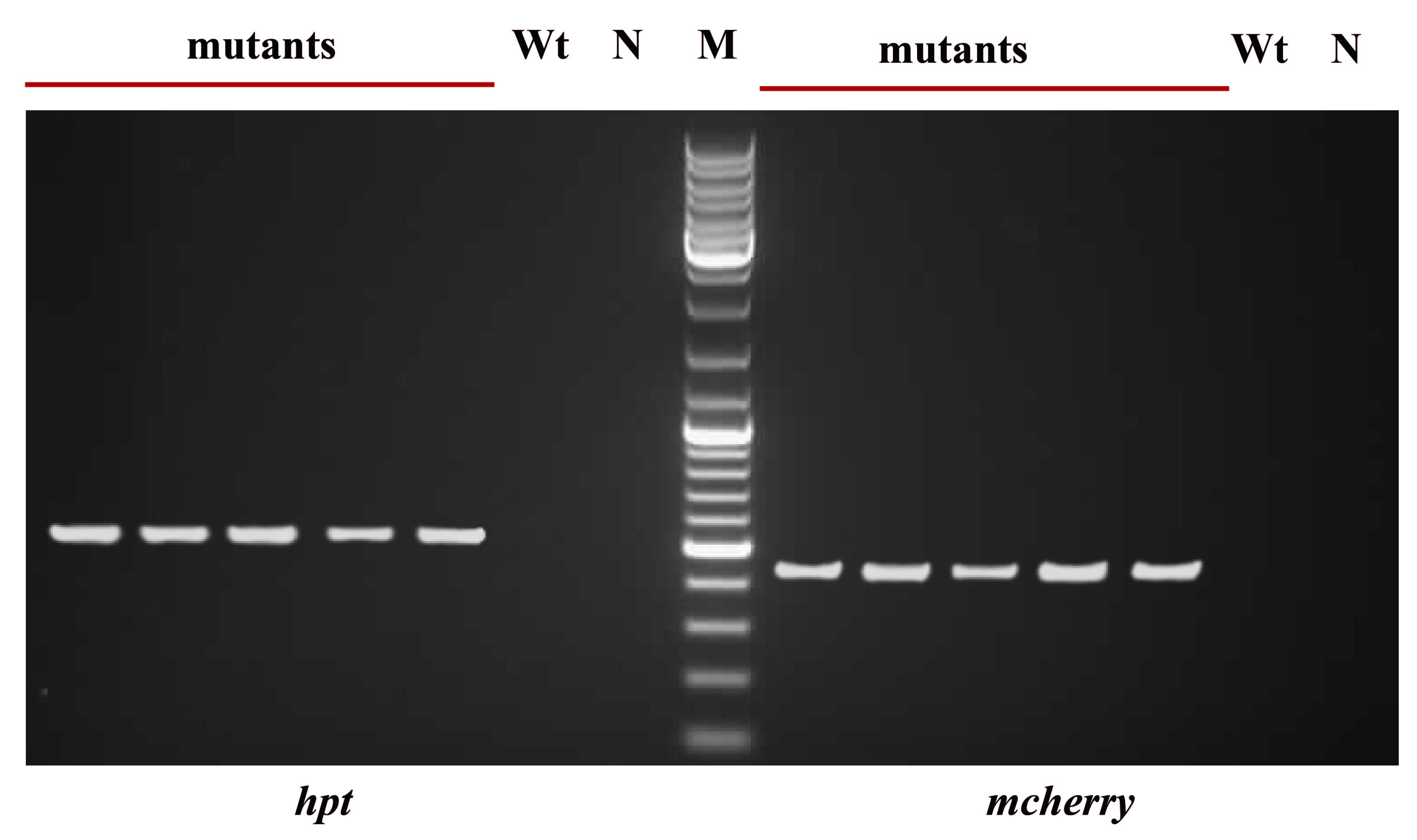

Supplement: Supplementary file 1 [file genes-09-00508-s001.zip › Supplementary File/Fig. S2.jpg]

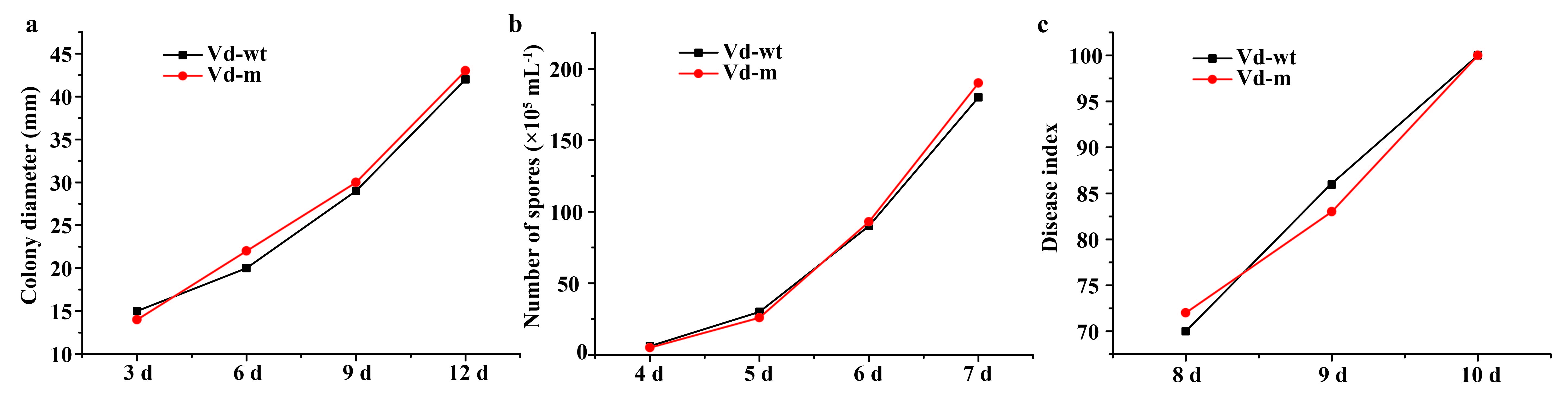

Supplement: Supplementary file 1 [file genes-09-00508-s001.zip › Supplementary File/Fig. S3.jpg]
